# Novel lignin α-O-4 derived hydrogen donors in CQ-based photoinitiating systems for dental resins

**DOI:** 10.1038/s41598-024-67377-z

**Published:** 2024-07-19

**Authors:** Lixia Xu, Ying Zhang, Shuqi Jin, Shuxin Luo, Kailun Chen, Sheng Fang, Liangjun Zhong, Jian Zhang, Rui He

**Affiliations:** 1 Center of Stomatology, The Affiliated Hospital of Hangzhou Normal University, Hangzhou, Zhejiang China; 2 The 3rd People’s Hospital of Deqing, Huzhou, Zhejiang China; 3 College of Material, Chemistry and Chemical Engineering, Key Laboratory of Organosilicon Chemistry and Material Technology, Ministry of Education, Hangzhou Normal University, Hangzhou, Zhejiang China; 4 Zhejiang Provincial Hospital of Chinese Medicine, Hangzhou, Zhejiang China; 5 School of Stomatology, Hangzhou Normal University, Hangzhou, Zhejiang China

**Keywords:** Photo-initiating system, Lignin α-O-4, Amine-free, Hydrogen donor, Dental resin, Photopolymerization, Materials science, Dentistry

## Abstract

The purpose of this work is to explore the properties of the lignin-derived amine-free photoinitiating systems (PISs) during the curing process. Four novel hydrogen donors (HD1, HD2, HD3, and HD4) derived from lignin α-O-4 structural were designed and synthesized by simple methods, and their low C–H bond dissociation energies on methylene were determined by molecular orbitals theory. Four experimental groups using CQ (camphorquinone)/HD PIs formulated with Bis-GMA/TEGDMA (70 w%/30 w%) were compared to CQ/EDB (ethyl 4-dimethylamino benzoate) system. The photopolymerization profiles and double bond conversion rate was tracked by FTIR experiments; the color bleaching ability of the samples and color aging test assay were performed using color indexes measurements; The cytotoxicity of the samples was also compared to EDB related systems. All of the experimental groups with new HDs were compared to the control group with EDB by statistical analysis. Compared to CQ/EDB system, new lignin-derived hydrogen donors combined with CQ showed comparable or even better performances in polymerization initiation to form resin samples, under a blue dental LED in air. Excellent color bleaching property was observed with the new HDs. Aging tests and cytotoxicity examination of the resin were performed, indicating the new lignin compounds to be efficient hydrogen donors for amine-free CQ-based photo-initiating system. Novel lignin α-O-4 derived hydrogen donors are promising for further usage in light-curing materials.

## Introduction

Light-curing resins are used extensively in material science such as coatings, inks, and 3D printing^1,2^, and played very important application in dentistry treatment such as dental composites and adhesives since 1960s^3^. Most of the light-curing resin materials for dentistry are consisted of monomer matrix such as bisphenol A-glycidyl methacrylate (Bis-GMA) of high viscosity and diluents such as triethylene glycol dimethacrylate (TEGDMA) of low viscosity, and amine/CQ-based photoinitiating system, as well as inorganic fillers^4^. Amine-containing photoinitiator plays a key role in initiating the chain reaction of monomers to achieve the light curing process in a short time, which still suffered from the following limitations^5–8^. Firstly, relatively low C=C bond conversion rate causes bio-safety problem by releasing unreacted monomers in a long-term. Secondly, unsatisfactory bleaching stability of the resin material by using amine-type photoinitiator will cause material discoloration and other aesthetic problems as well. Therefore, novel photoinitiators with low toxicity, high C=C bond conversion, and good bleaching properties are highly desirable^9–12^. Recently, Lalevée and co-workers developed sesamol-, 6-hydroxy-3-coumaranone- and 5-hyrdoxy-2(3 H)-benzofuranone derived novel co-initiators for amine-free CQ-based ternary systems^10^. The natural curcumin and 4-methylumbelliferone derived acrylates^12^, as well as aromatic amine derived acrylate^11^ were also demonstrated to be highly efficient hydrogen donors. The same group also introduced sulfinates and sulfonates as co-initiators in CQ-based photoinitiating system^9^.

Lignin is a highly abundant aromatic polymer in nature, and its chemical conversion has attracted many attentions^13^. Lignin is consisted of diverse monomeric units such as β-O-4, α-O-4, 4-O-5, and β-1 linkages^14^. Many efforts have been made to the chemical conversion of β-O-4 linkage which derived from lignin by degradation^15,16^, however, there are rare reports on utilization of α-O-4 lignin linkage^17^. To the best of our knowledge, there is no report on utilization of lignin linkage, such as lignin-based photoinitiating system, in the field of dental material^18^.

**Figure 1:**
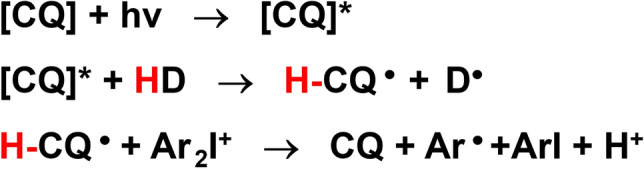
Reaction mechanism for the new hydrogen donors in CQ-based photoinitiating polymerization.

**Figure 2:**
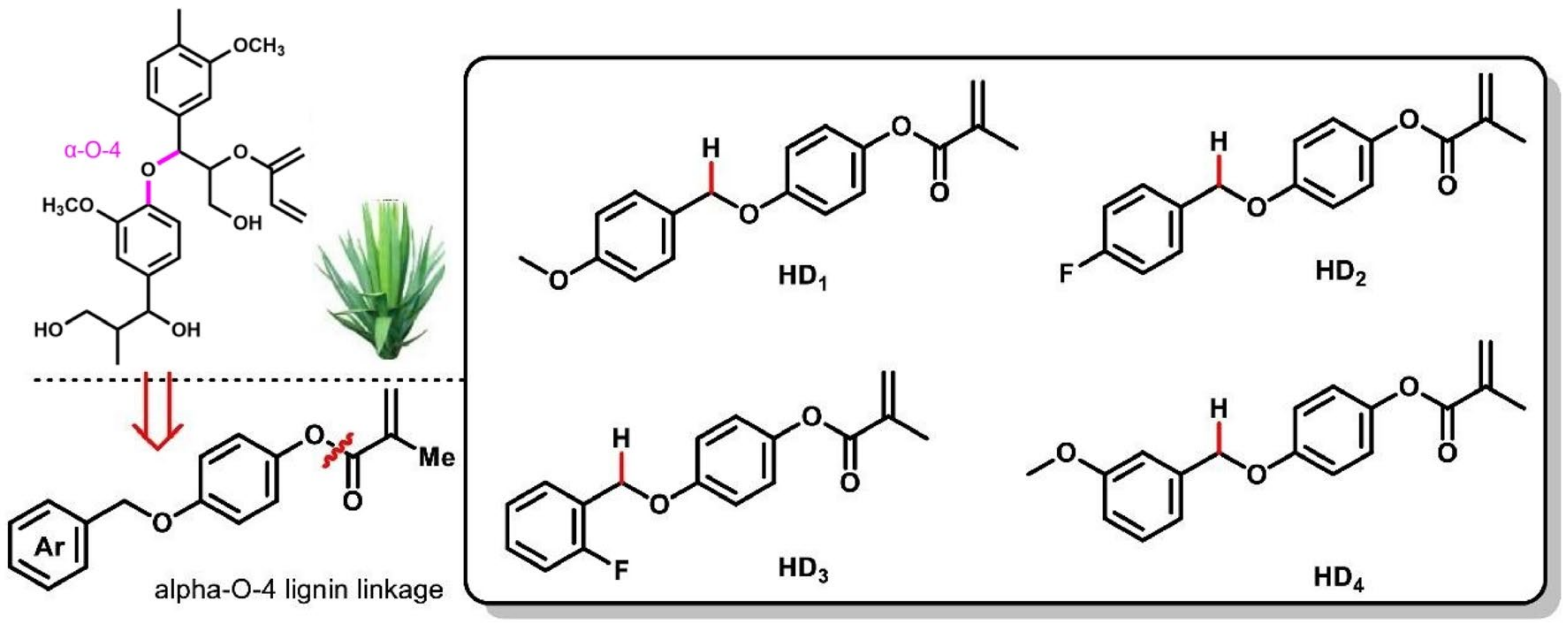
HD1, HD2, HD3 and HD4 derived from α-O-4 lignin model.

Based on our previous work on lignin conversion and with our continuing interest on lignin utilization^19–22^, we hypothesized that α-O-4 lignin derivative could be efficient hydrogen donor in Type II photoinitiating polymerization due to the presence of a labile hydrogen bearing on the methylene, which is supported by C–H Bond Dissociation Energy (BDE) calculations. In the photoinitiating system, when the photoinitiator such as CQ is excited by light, these hydrogen donors provide hydrogen atoms to generate free radicals, which initiate the polymerization of methacrylate monomers (Fig. 1). The polymerizable groups on these donors reduce their migration and leaching from the polymer, enhancing the efficiency and stability of the system. By introducing a copolymerizable part of methacrylate, these new co-initiators also act as a polymerizable monomer and are therefore much less subject to migration. Herein, we designed and synthesized four new co-initiators HD1, HD2, HD3 and HD4 (HDs) derived from α-O-4 lignin model by simple chemical synthesis (Fig. 2). It is demonstrated that the HD/CQ/Iod [Bis (4-tert-butylphenyl) iodonium hexafluorophosphate)] photoinitiator systems exhibit superior polymerization performance and biocompatibility compared to the traditional EDB/CQ system.

## Materials and methods

### Compounds

**Figure 3:**
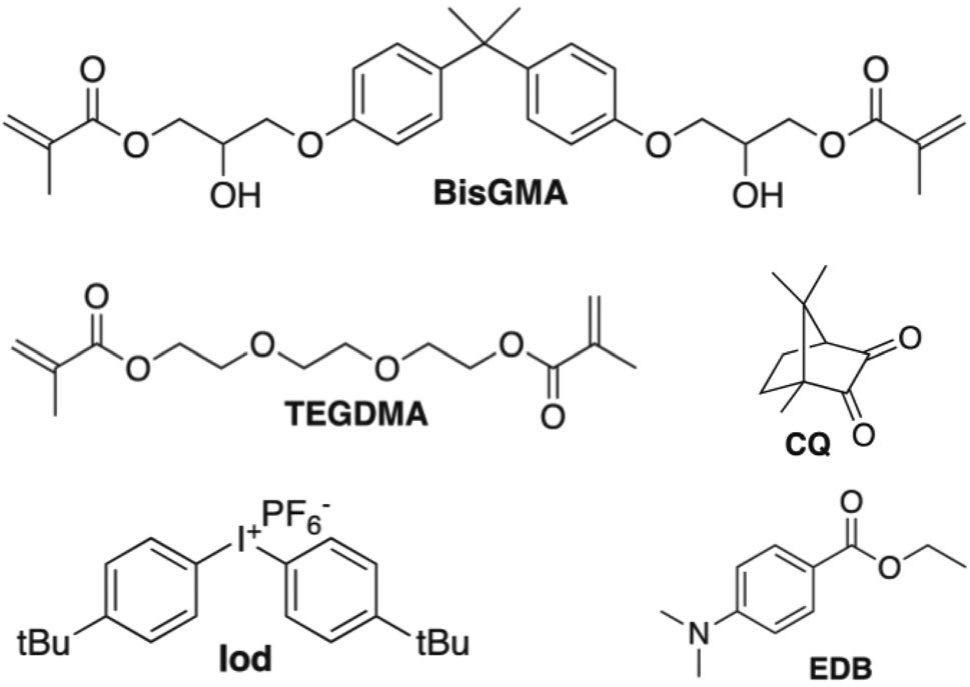
Chemical structures of other components of photoinitiating systems.

All of the chemical materials were used as received by manufacturers. Bis-GMA was obtained from Sigma Aldrich (CA, USA, Fig. 3). TEGDMA, CQ, Iod and ethyl 4-diethylaminobenzoate (EDB) were obtained from TCI (Tokyo, Japan, Fig. 3). Novel hydrogen donors (HD1, HD2, HD3 and HD4) were obtained from acylation of 4-methoxybenzyl alcohol (TCI, Tokyo, Japan), 4-fluorobenzyl alcohol (TCI, Tokyo, Japan), 2-fluorobenzyl alcohol (TCI, Tokyo, Japan) and *m*-methoxybenzyl alcohol (TCI, Tokyo, Japan) according to synthetic procedure described below.

^1^H-NMR spectra were recorded at room temperature on a Varian 500 MHz spectrometer, and data for ^1^H-NMR spectra are reported as following: chemical shift (multiplicity, coupling constants, integrations). Abbreviations are as follows: s (singlet), d (doublet), m (multiplet). Solvent used in NMR techniques was Chloroform-D1.

### Syntheses of HD1, HD2, HD3 and HD4

**Figure 4:**
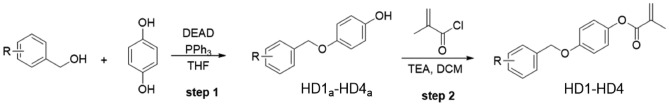
Synthetic route toward hydrogen donor based on lignin α-O-4 model.

In this study, lignin α-O-4 hydrogen donors were obtained by the following chemical synthesis (Fig. 4).

**Figure 5:**
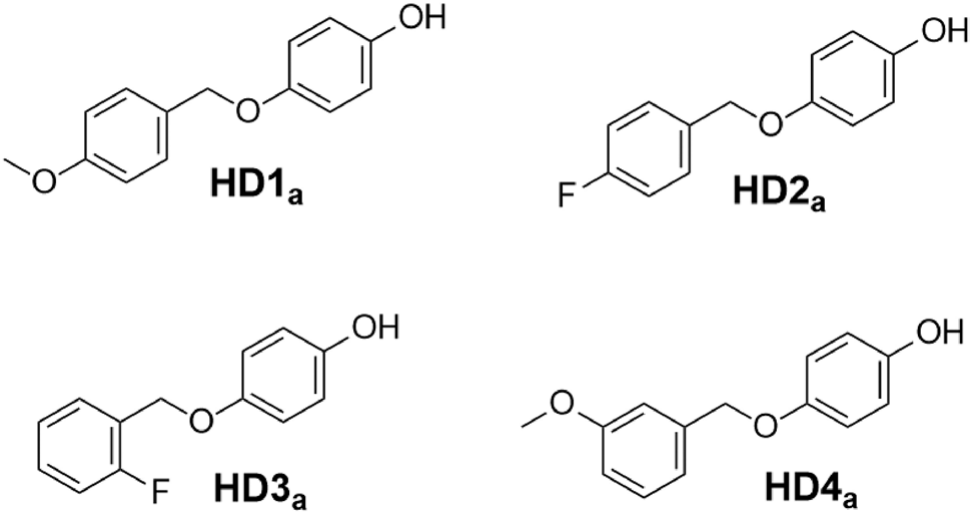
Structural formula of HDs_a_.

**Figure 6:**
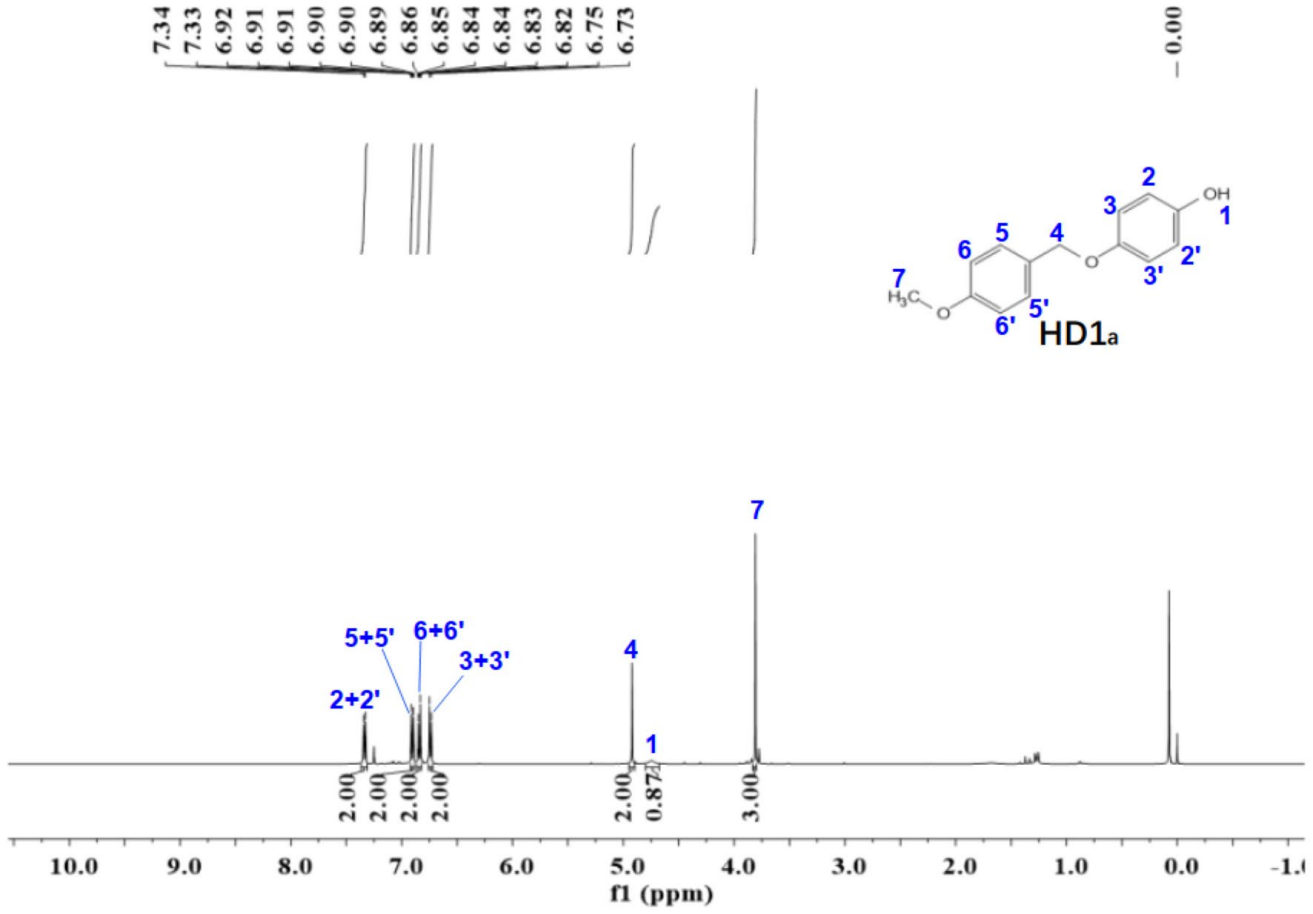
The ^1^H-NMR spectra of HD1_a_.

**Figure 7:**
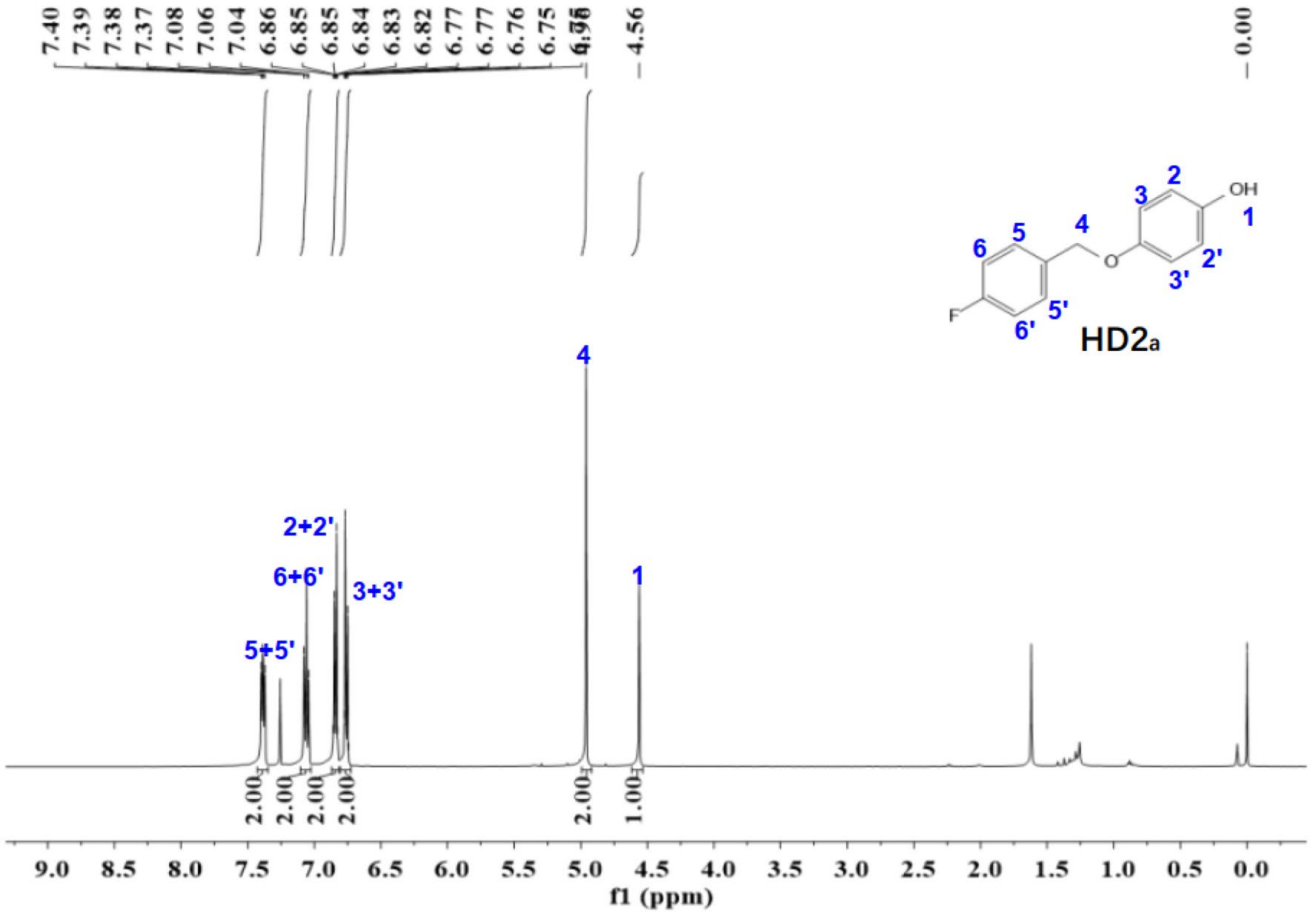
The ^1^H-NMR spectra of HD2_a_.

**Figure 8:**
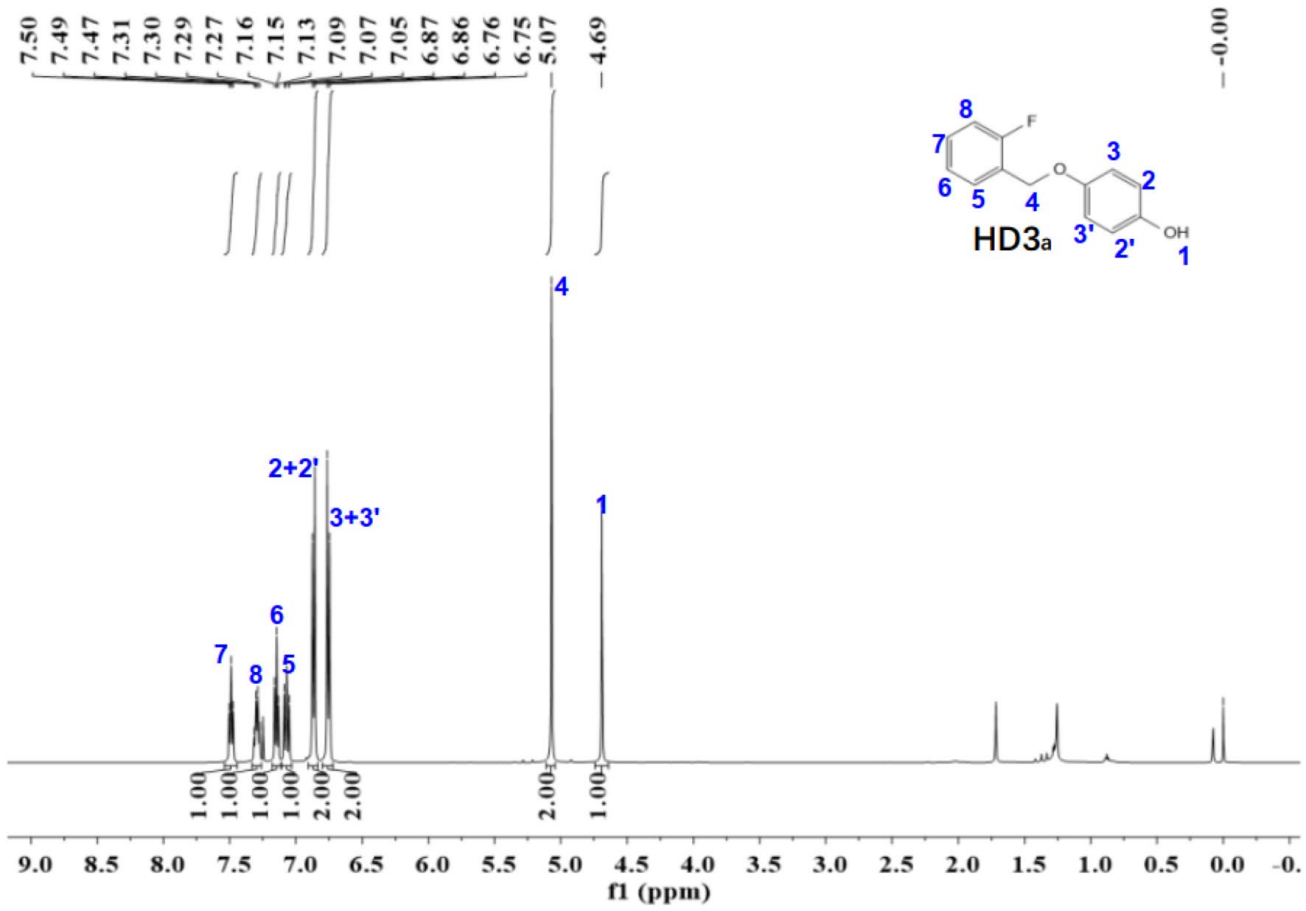
The ^1^H-NMR spectra of HD3_a_.

**Figure 9:**
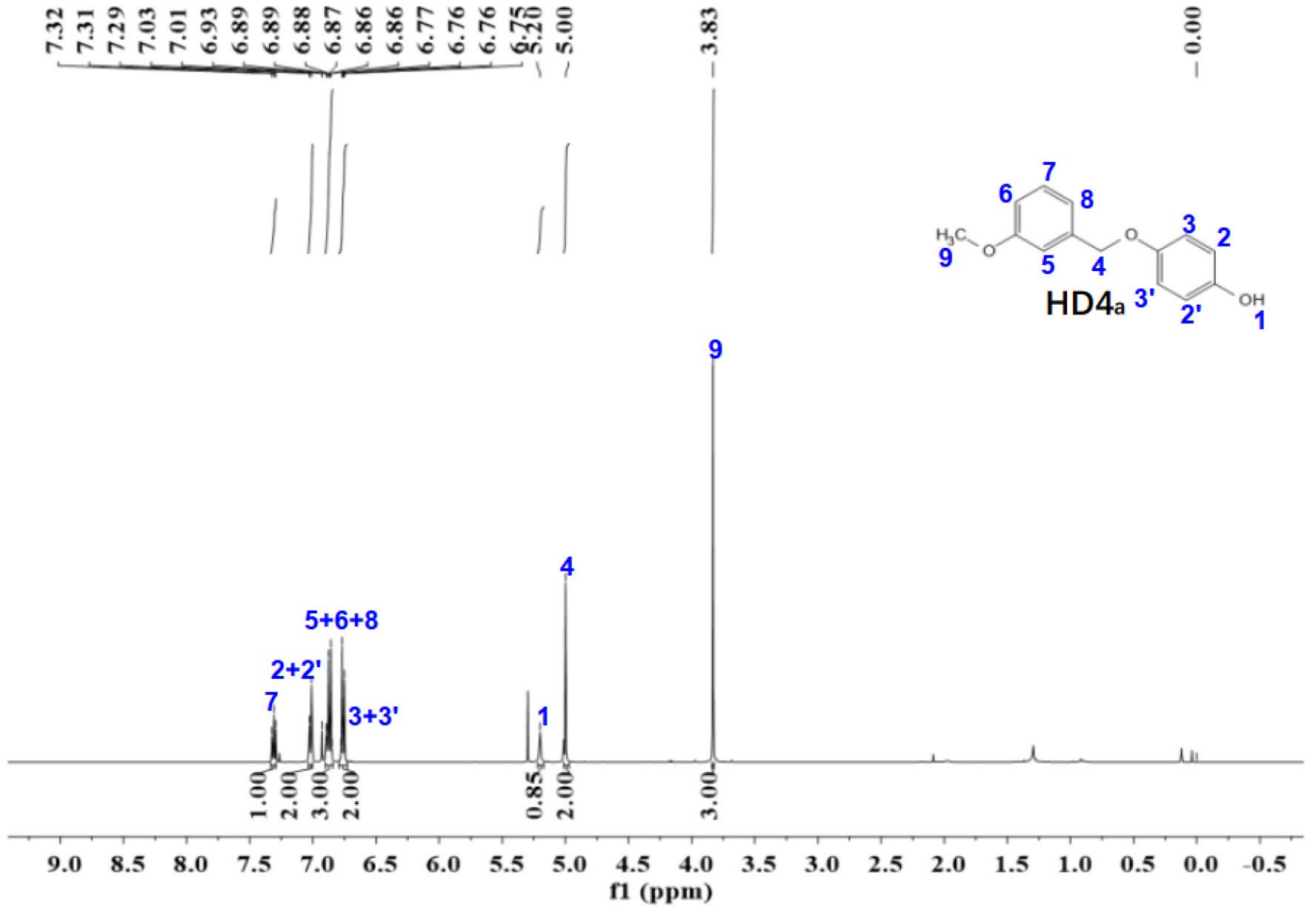
The ^1^H-NMR spectra of HD4_a_.

**Step 1, General steps for the synthesis of HDs**_**a**_. Benzyl alcohol (1.0 equiv.), hydroquinone (2.0 equiv.) and triphenylphosphine (PPh_3_) (2.0 equiv.) were dissolved in tetrahydrofuran (THF) in sequence. After cooling to 0 °C, diethyl azodicarboxylate (DEAD) (2.0 equiv.) was added dropwisely. The reaction was kept at 0 °C for about 10 min and then warmed to room temperature with stirring for overnight. At the end of the reaction, water was added to quench the reaction and the organic phase was washed with water, dried over anhydrous MgSO_4_, filtered and concentrated under reduce pressure. The reaction mixture was purified by chromatography on silica gel using EA/PE (ethyl acetate/petroleum ether) (1/3, v/v) as eluent to give compounds HDs_a_ (Fig. 5). The ^1^H-NMR spectra of HDs_a_ as follows (Figs. 6, 7, 8, 9). The ^13^C-NMR spectra of HDs_a_ are included in supplementary material (Supplementary Figs. 1–4).

HD1_a_ was obtained according to the general step 1 using 4-methoxybenzyl alcohol (276 mg, 2 mmol), hydroquinone (440 mg, 4 mmol), PPh_3_ (1049 mg, 4 mmol) and DEAD (697 mg, 2 mmol). After purification of the crude reaction mixture by chromatography on silica gel using EA/PE, the product was obtained as a crystalline peach solid, 217 mg, yield = 47%.

The ^1^NMR spectrum of HD1 is shown in Fig. 6. ^1^H NMR (500 MHz, chloroform-d) δ 7.34 (d, *J* = 8.7 Hz, 2H), 6.93–6.88 (m, 2H), 6.86–6.82 (m, 2H), 6.76–6.72 (m, 2H), 4.92 (s, 2H), 4.75 (s, 1H), 3.81 (s, 3H).

HD2_a_ was obtained according to the general step 1 using 4-fluorobenzyl alcohol (252 mg, 2 mmol), hydroquinone (440 mg, 4 mmol), PPh_3_ (1049 mg, 4 mmol) and DEAD (697 mg, 4 mmol). After purification of the crude reaction mixture by chromatography on silica gel using EA/PE, the product was obtained as a white solid, 251 mg, yield = 58%.

The ^1^NMR spectrum of HD1 is shown in Fig. 7. ^1^H NMR (500 MHz, chloroform-d) δ 7.38 (dd, *J* = 8.3, 5.5 Hz, 2H), 7.06 (t, *J* = 8.7 Hz, 2H), 6.87 – 6.82 (m, 2H), 6.79 – 6.72 (m, 2H), 4.96 (s, 2H), 4.56 (s, 1H).

HD3_a_ was obtained according to the general step 1 using 2-fluorobenzyl alcohol (252 mg, 2 mmol), hydroquinone (440 mg, 4 mmol), PPh_3_ (1049 mg, 4 mmol) and DEAD (697 mg, 4 mmol). After purification of the crude reaction mixture by chromatography on silica gel using EA/PE, the product was obtained as a white solid, 208 mg, yield = 47%.

The ^1^NMR spectrum of HD1 is shown in Fig. 8. ^1^H NMR (500 MHz, Chloroform-d) δ 7.49 (t, *J* = 7.3 Hz, 1H), 7.29 (q, *J* = 7.3, 6.7 Hz, 1H), 7.15 (t, *J* = 7.4 Hz, 1H), 7.07 (t, *J* = 9.2 Hz, 1H), 6.87 (d, *J* = 8.9 Hz, 2H), 6.76 (d, *J* = 8.9 Hz, 2H), 5.07 (s, 2H), 4.69 (s, 1H).

HD4_a_ was obtained according to the general step 1 using *m*-methoxybenzyl alcohol (276 mg, 2 mmol), hydroquinone (440 mg, 4 mmol), PPh_3_ (1049 mg, 4 mmol) and DEAD (697 mg, 4 mmol). After purification of the crude reaction mixture by chromatography on silica gel using EA/PE, the product was obtained as a pale yellow solid, 239 mg, yield = 52%.

The ^1^NMR spectrum of HD1 is shown in Fig. 9. ^1^H NMR (500 MHz, Chloroform-d) δ 7.31 (t, *J* = 7.8 Hz, 1H), 7.02 (d, *J* = 8.0 Hz, 2H), 6.87 (d, *J* = 10.6, 3.7, 3.0 Hz, 3H), 6.79 – 6.73 (m, 2H), 5.20 (s, 1H), 5.00 (s, 2H), 3.83 (s, 3H).

**Figure 10:**
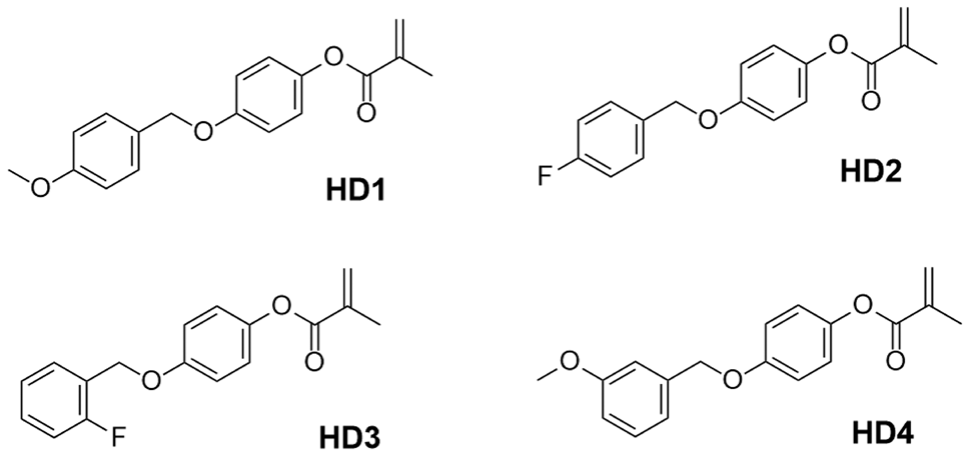
Structural formula of HDs.

**Figure 11:**
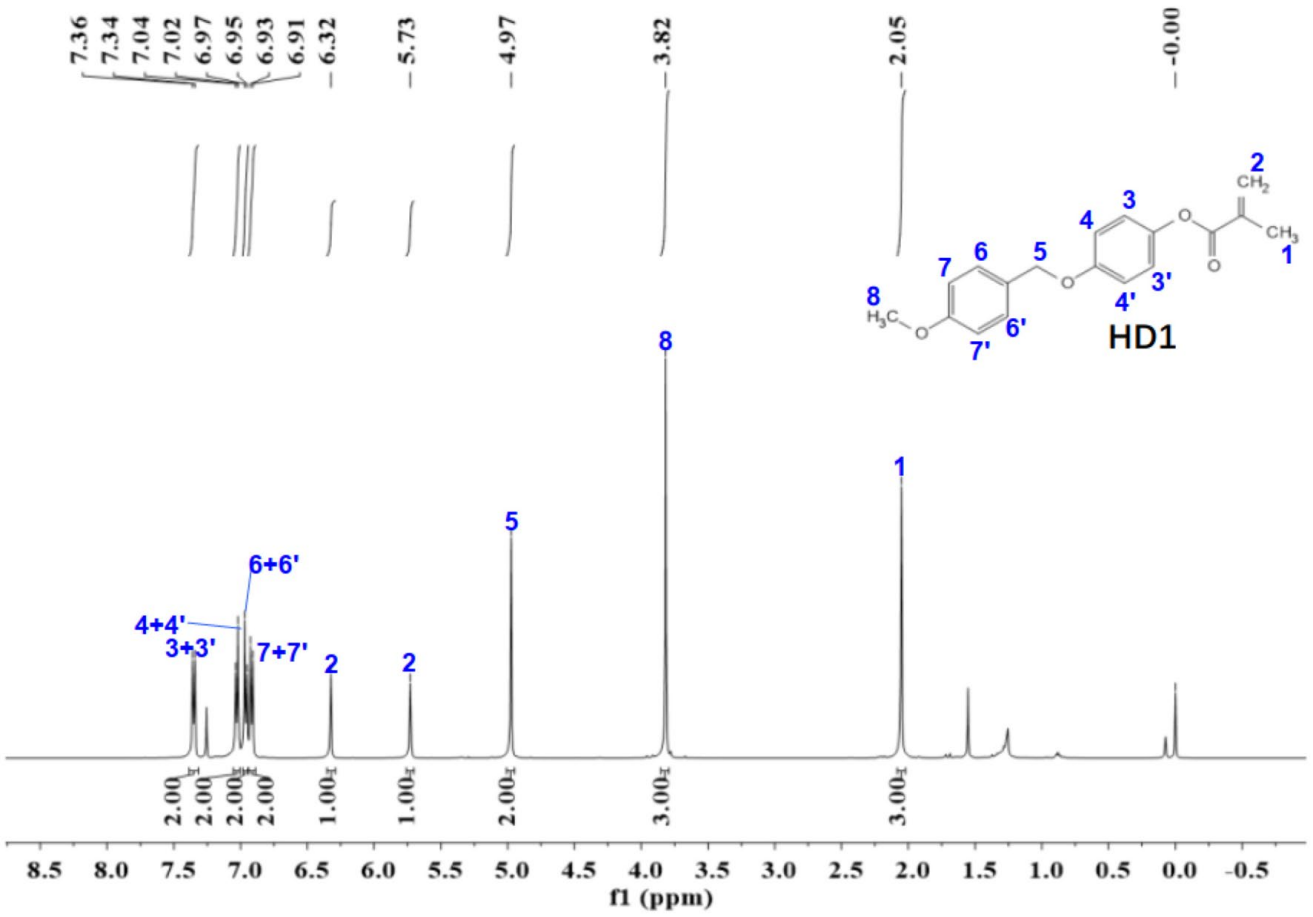
The ^1^H-NMR spectra of HD1.

**Figure 12:**
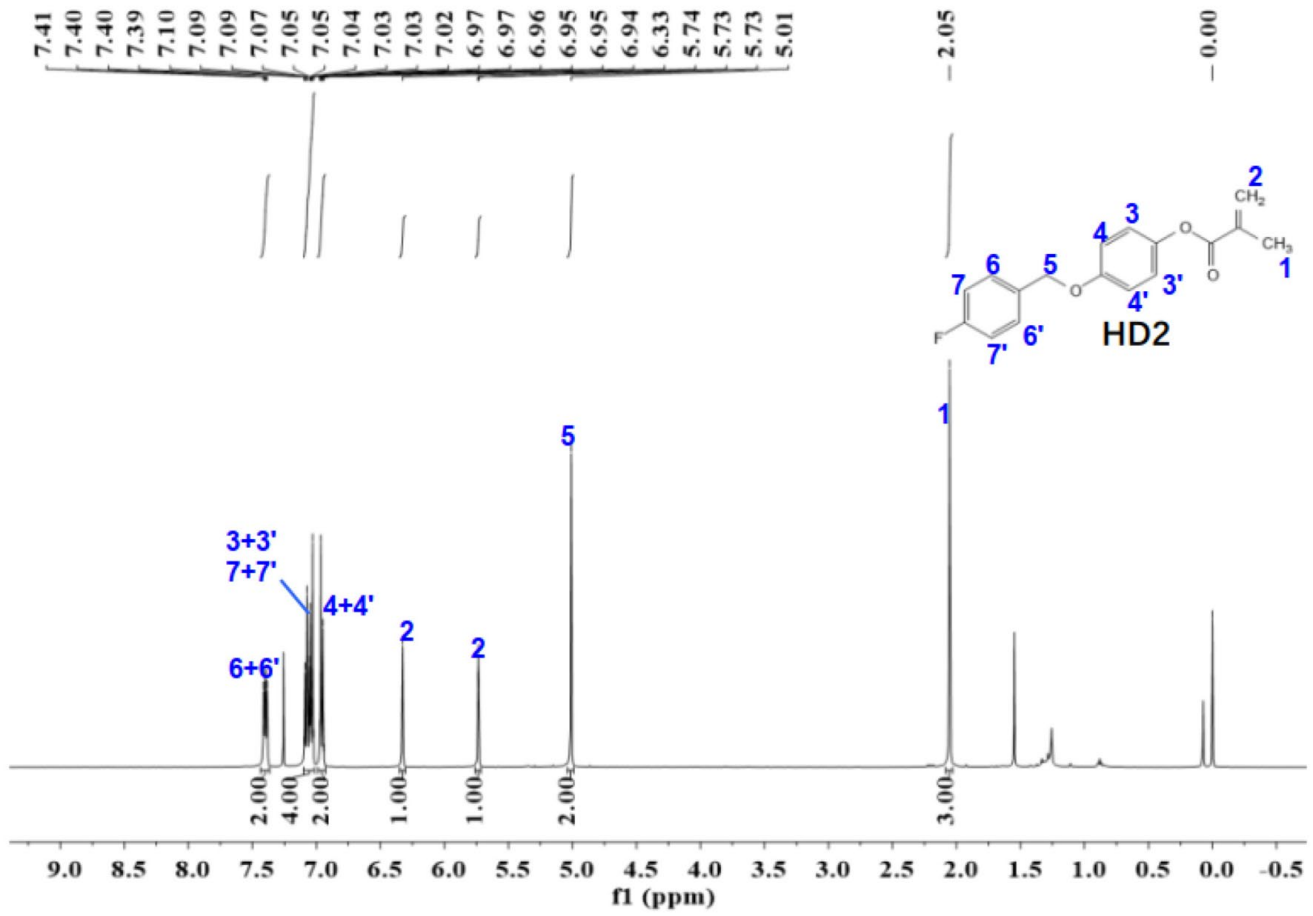
The ^1^H-NMR spectra of HD2.

**Figure 13:**
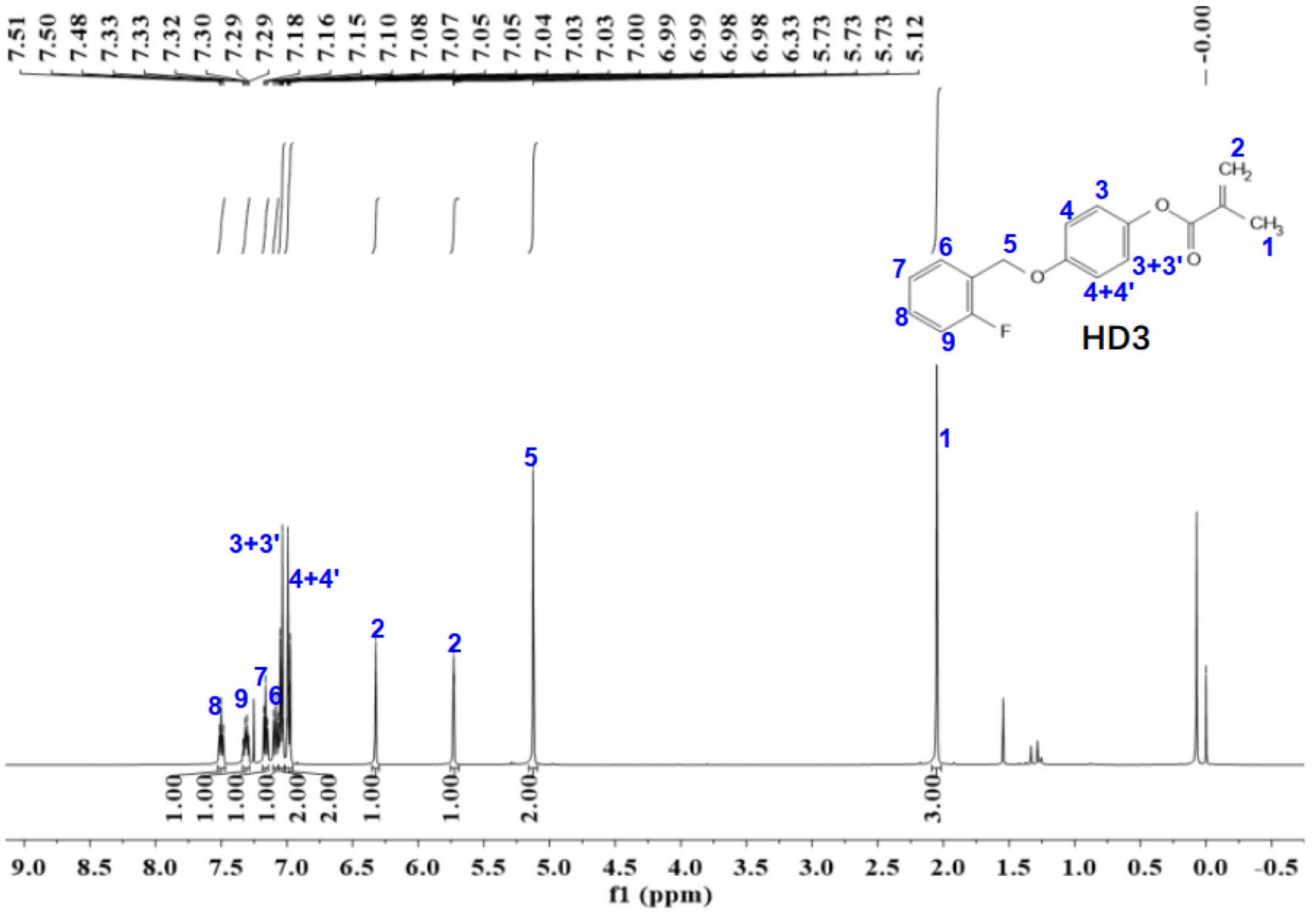
The ^1^H-NMR spectra of HD3.

**Figure 14:**
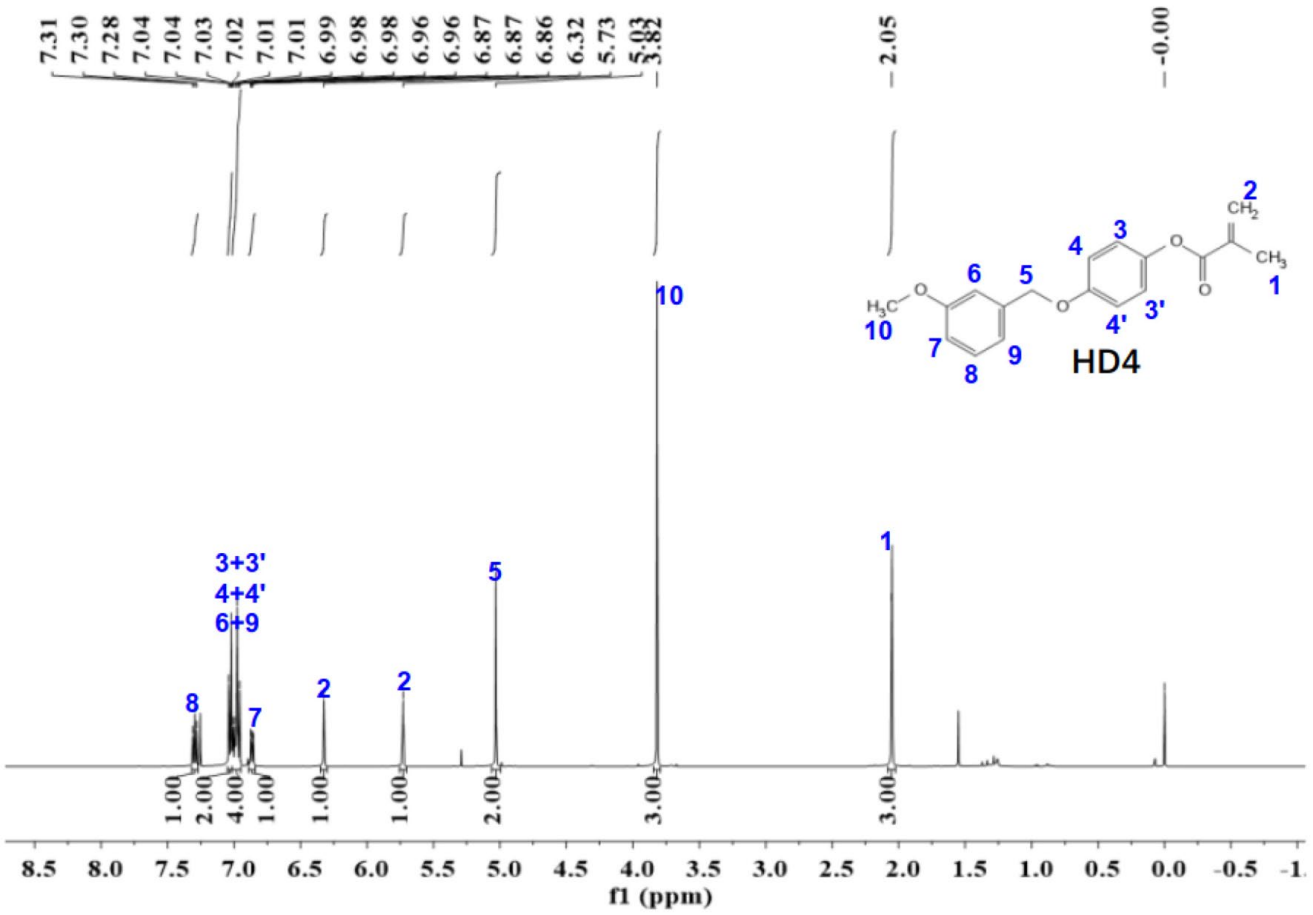
The ^1^H-NMR spectra of HD4.

**Step 2**, **General synthesis of HDs**. HD_a_ (1 eq) and triethylamine (TEA, 1.2 eq) were dissolved in dichloromethane (DCM), and acyl chloride (1.2 eq) was added in dropwise at 0 °C. The reaction was stirred at 0 °C for 10 min and then warmed to room temperature with stirring for overnight. At the end of the reaction, the reaction was quenched with water and the organic phase was washed with water, dried over MgSO_4_, and filtered and concentrated under reduced pressure. The reaction mixture was purified by chromatography on silica gel by chromatography on silica gel using EA/PE (1/15, v/v) as eluent to give compounds HDs (Fig. 10). The ^1^H-NMR spectra of HDs as follows (Figs. 11, 12, 13, 14). The ^13^C-NMR spectra of HDs have been included in supplementary material (Supplementary Figs. 5–8).

HD1 was prepared from HD1_a_ (217 mg, 0.94 mmol), acyl chloride (171 mg, 1.04 mmol), TEA (114 mg, 1.13 mmol) according to the general step 2. After purification of the crude reaction mixture by chromatography on silica gel using EA/PE, the product was obtained as a white solid, 172 mg, yield = 62%.

The ^1^NMR spectrum of HD1 is shown in Fig. 11. ^1^H NMR (500 MHz, chloroform-d) δ 7.35 (d, *J* = 8.5 Hz, 2H), 7.03 (d, *J* = 9.0 Hz, 2H), 6.96 (d, *J* = 9.0 Hz, 2H), 6.92 (d,* J* = 8.6 Hz, 2H), 6.32 (s, 1H), 5.73 (s, 1H), 4.97 (s, 2H), 3.82 (s, 3H), 2.05 (s, 3H).

HD2 was prepared from HD2_a_ (1131 mg, 5.18 mmol), acyl chloride (938 mg, 5.70 mmol), TEA (629 mg, 6.22 mmol) according to the general step 2. After purification of the crude reaction mixture by chromatography on silica gel using EA/PE, the product was obtained as a light-yellow solid, 1213 mg, yield = 78%.

The ^1^NMR spectrum of HD1 is shown in Fig. 12. ^1^H NMR (500 MHz, chloroform-d) δ 7.40 (dd,* J* = 8.5, 5.4 Hz, 2H), 7.11–7.01 (m, 4H), 7.00–6.93 (m, 2H), 6.33 (s, 1H), 5.77–5.70 (m, 1H), 5.01 (s, 2H), 2.05 (s, 3H).

HD3 was prepared from HD3_a_ (208 mg, 0.95 mmol), acyl chloride (172 mg, 1.05 mmol), TEA (115 mg, 1.14 mmol) according to the general step 2. After purification of the crude reaction mixture by chromatography on silica gel using EA/PE, the product was obtained as a light-yellow solid, 251 mg, yield = 88%.

The ^1^NMR spectrum of HD1 is shown in Fig. 13. ^1^H NMR (500 MHz, chloroform-d) δ 7.50 (t, *J* = 7.5 Hz, 1H), 7.34–7.28 (m, 1H), 7.16 (t, *J* = 7.5 Hz, 1H), 7.11–7.06 (m, 1H), 7.06–7.02 (m, 2H), 7.01–6.97 (m, 2H), 6.33 (s, 1H), 5.75–5.69 (m, 1H), 5.12 (s, 2H), 2.05 (d, *J* = 1.2 Hz, 3H).

HD4 was prepared from HD4_a_ (239 mg, 1.04 mmol), acyl chloride (188 mg, 1.14 mmol), TEA (126 mg, 1.25 mmol) according to the general step 2. After purification of the crude reaction mixture by chromatography on silica gel using EA/PE, the product was obtained as a white solid, 242 mg, yield = 75%.

The ^1^NMR spectrum of HD1 is shown in Fig. 14. ^1^H NMR (500 MHz, chloroform-d) δ 7.30 (t, *J* = 7.9 Hz, 1H), 7.02 (td, *J* = 6.8, 3.0 Hz, 2H), 7.00–6.95 (m, 4H), 6.86 (dd, *J* = 8.2, 2.3 Hz, 1H), 6.32 (s, 1H), 5.75–5.71 (m, 1H), 5.03 (s, 2H), 3.82 (s, 3H), 2.05 (s, 3H).

### Irradiation sources

Photopolymerization of the resin samples was irradiated by a blue LED@400–490 nm which is commonly used in dental materials (FUSION from Dent Light ~ 1500 mW/cm^2^).

### Photopolymerization experiments

#### Preparation of the photosensitive formulations

Mixture of Bis-GMA and TEGDMA (70%/30% w/w) was stirred for 30 min and used as resin matrix. Then CQ, Iod, EDB or HDs were added as a photoinitiating system into resin matrix and stirred for 2 h at RT in the dark before usage. Comparison group is consisted of 69.3 wt% Bis-GMA, 29.7 wt% TEGDMA, 0.5 wt% CQ, and 0.5 wt% EDB (group EDB-1); comparison group 2 is consisted of 68.6 wt% Bis-GMA, 29.4 wt% TEGDMA, 0.5 wt% CQ, 0.5 wt% EDB and 1 wt% Iod (group EDB-2); experiment group is consisted of 68.25 wt% Bis-GMA, 29.25 wt% TEGDMA, 0.5 wt% CQ, 1 wt% Iod and 1 wt% HDs^10,23^ (group HDs).

#### Photopolymerization experiments

Vertex-70 (Bruker, German) was used to detect the C=C bond content of the mixture during the light curing process, and the mixture prepared according the method above was dropped on the carrier table of Vertex-70, controlling the curing time for Droplet-shaped sample by FUSION, scanned. The instrument scans every second for the first 20 s of the curing process, and then at 30 s, 40 s, 50 s, 60 s, 80 s, 100 s, and 120 s thereafter. The C=C bond conversion rate was calculated by comparing the area of the wave peaks^24^ at 1608 cm^−1^ and 1635 cm^−1^ (N = 3).

The double bond conversion rate (DC) was calculated using the following formula:

$${\text{DC}} = \left[ {1 - \left( {{\text{S}}_{{\text{c = c}}} /{\text{S}}_{{{\text{ph}}}} } \right)_{{\text{a}}} /\left( {{\text{S}}_{{\text{c = c}}} /{\text{S}}_{{{\text{ph}}}} } \right)_{{\text{b}}} } \right] \, \times \, 100\%$$

The polymerization rate (Rp) was calculated using the following formula:

$${\text{Rp }} = {\text{ DC}}\;({\text{t}}) \, - {\text{ DC}}\;({\text{t}} - 1)$$

where S_C=C_ is the absorbance intensity of methacrylate C=C at 1635 cm^−1^, and S_ph_ is phenyl ring at 1608 cm^−1^; (S_C=C_/S_ph_)_b_ is the normalized absorbance of the functional group before being irradiated, and (S_C=C_/S_ph_) _a_ is after being irradiated.

### Colorimetric measurements

Transmission experiments were performed using datacolor 1050 to determine the L*, a*, b* colorimetric values of the obtained dental resins. Each group includes 3 resin specimens sized with a diameter of 1.5 cm and a thickness of 0.1 cm. After each sample was fully cured for the same duration, they were subjected to analysis using Datacolor 1050, and any differences in color were statistically examined (N = 3).

### Color stability

The color stability of the samples was examined using a method based on ISO 4049:2009/ISO 7491 guideline with a slight modification. Each group includes 3 resin specimens which were sized with a diameter of 1.5 cm and a thickness of 0.1 cm, prepared by using the FUSION, and initial L*, a* and b* values of each specimen were determined according to the following ageing scenario by using a Datacolor 1050. In scenario 1, the disc sample was kept in the dark and dry in an oven at 37 ± 2 °C for 7 days. In scenario 2, the disc sample was stored in the dark and in water inside an oven at 37 ± 2 °C for 7 days. The third sample was kept in dark and dry inside an oven at 37 ± 2 °C for 24 ± 2 h, which was then taken out from the oven and a half was blanked off using aluminum foil (for Scenario 3a: uncovered side; for Scenario 3b: covered side). After that, the sample was immersed in water (37 ± 2 °C) (water level was 10 ± 3 mm above the sample) and exposed to the radiation for 24 h at 150,000 ± 15,000 Lux in a radiation chamber. Subsequently, the aluminum foil on the specimen was removed and the sample was stored in dark and dry at 37 ± 2 °C inside the oven for 5 days. The above ageing scenario was performed for each specimen to measure color (ΔE) change using a Datacolor 1050^9^ (N = 3).

The ΔE value for samples after ageing scenario was calculated by using the following Eq. (1):

1 $$\Delta {\text{E}} = {\text{ delta}}\;{\text{E}} = \sqrt {\Delta {\text{L}} ^{*2} + \Delta {\text{a}}^{*2} + \Delta {\text{b}}^{*2} }$$

### Cytotoxicity evaluation

Each group includes 8 resin specimens and each specimen was sized with a diameter of 0.6 cm and a thickness of 0.2 cm, affording a total surface area of 7.54 cm^2^. The eluate from resin specimens was obtained according to the ISO10993-12 international standard.

The testing specimens were firstly sterilized with a UV lamp for 30 min, and then were placed in a centrifuge tube with 6.03 mL of DMEM (Cellmax, China) as medium, with a surface area of specimens/volume of medium = 1.25 cm^2^/mL. The sample in DMEM was stored at 37 °C for 24 h and the eluate was collected for the further test^25^.

The L929 mouse fibroblast cells (Cell Bank of the Chinese Academy of Sciences, Shanghai, China) were seeded in 96-well plates at a density of 2000 cells/well. While the experimental group was cultured using respective eluate, the control group was cultured using DMEM medium including 10% fetal bovine serum (Cellmax, China). The following detection of cytotoxicity was performed using CCK-8 reagent (Beyotime Biotechnology, China), and a microplate reader (synergy H1, Bio Tek, Vermont, USA) was employed to measure the optical density (OD) at 450 nm after incubation for 24 h, 72 h, and 120 h. Wells without cells but with the CCK-8 solution were used as the blank control group (N = 5).

$${\text{The RGR}} = \left( {{\text{OD of the experimental group}} - {\text{OD of the blank group}}} \right)/\left( {{\text{OD of the control group}} - {\text{OD of the blank group}}} \right) \times {1}00\% .$$

### Mechanical properties

The elastic modulus and flexural strength tests were conducted using a universal testing machine (LiShi, Shanghai). Each group consisted of 3 samples, with each sample measuring 2 mm × 2 mm × 80 mm (adjusted according to ISO 4049). Each specimen was cured in segments for 40 s per area to ensure complete curing, and a three-point bending test was performed using the universal testing machine.

### Molecular modelling

The benzylic C–H bond dissociation energies for HDs were calculated at the M06-2X1/6-311G (d, p) level using the Gaussian 16 program package^26^.

### Statistical analysis

Statistical analysis was performed using GraphPad Prism software. The experiments were repeated three times, the results are presented as the mean ± standard error of the mean (mean ± SEM), one-way analysis of variance (ANOVA) was used for data analysis. A P-value of ≤ 0.05 was considered statistically significant.

## Results

### Photopolymerization experiments

**Figure 15:**
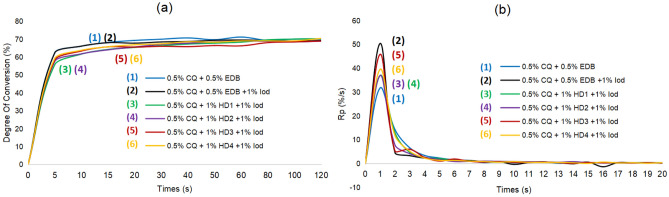
Photopolymerization profiles for different system based on lignin α-O-4 hydrogen donor compared to system based on EDB. (**a**) Degree of C=C conversion vs. time of HDs; (**b**) rate of polymerization vs. time of HDs.

**Figure 16:**
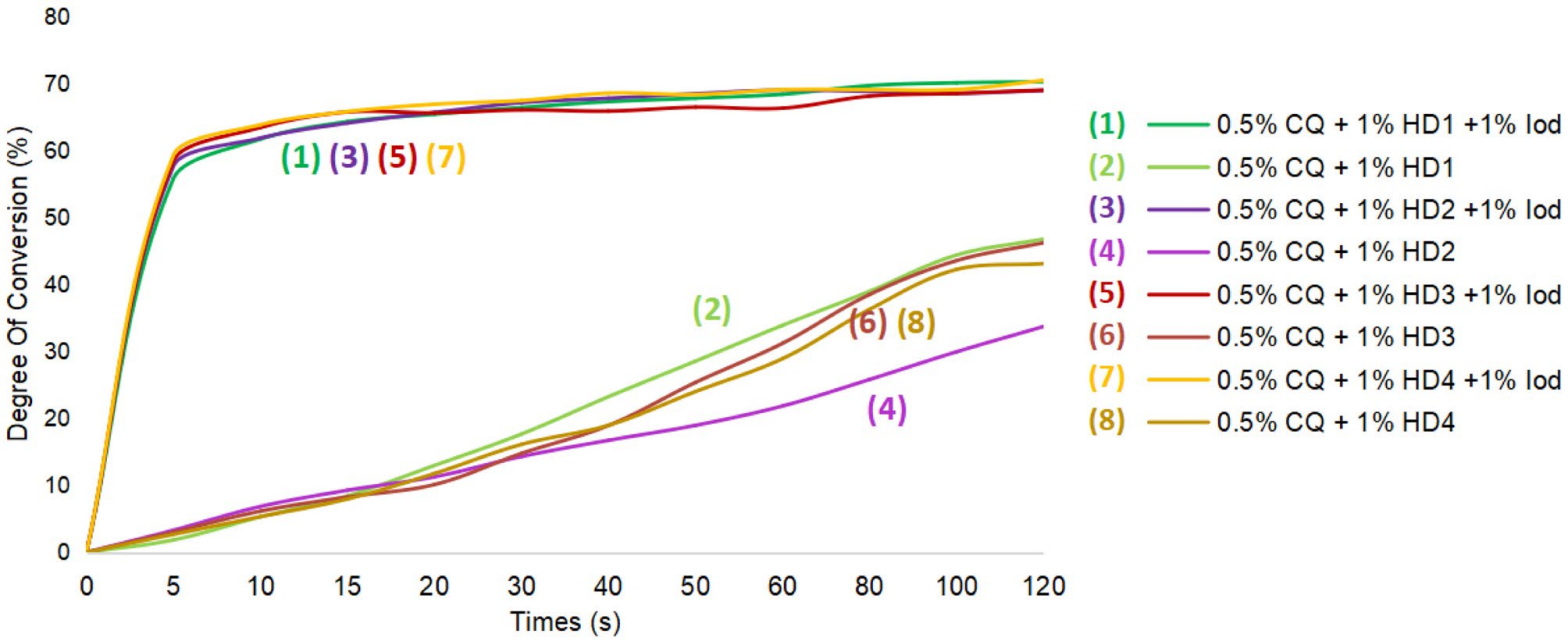
Photopolymerization profiles for different HDs with Iod or without Iod.

As described in Fig. 15, ternary systems consisted of CQ/HD/Iod exhibited comparable and statistically similar results with the CQ/EDB and CQ/EDB/Iod system in photopolymerization after 120 s, all of which afforded ~ 70% DC (degree of conversion) (HD1 ~ 70.23%, HD2 ~ 68.91%, HD3 ~ 68.97%, HD4 ~ 70.42% vs. EDB-1 ~ 70.05%, EDB-2 ~ 69.28%) (statistical results are shown in Table 1). Photo-initiating systems without Iod led to significantly lower DC at 20 s (HD1 ~ 12.96%, HD2 ~ 11.25%, HD3 ~ 10.10%, HD4 ~ 11.75%) and 120 s (HD1 ~ 46.74%, HD2 ~ 33.69%, HD3 ~ 46.19%, HD4 ~ 43.07%) (Fig. 16).

**Table 1:** DC and Rp_max_ for CQ/HDs/Iod and CQ/EDB systems (N = 3).

	DC at 20 s (%) (mean ± SEM %)	DC at 120 s (%) (mean ± SEM %)	Rp_max_ (%/s) (mean ± SEM %/s)
EDB-1	69.31 ± 0.9121	70.05 ± 1.1850	31.75 ± 1.2030
EDB-2	65.61 ± 2.469	69.28 ± 2.145	50.40 ± 5.264
HD1	65.38 ± 0.3341	70.23 ± 0.7912	36.99 ± 1.2850^d^
HD2	65.63 ± 0.2157	70.23 ± 1.8110	36.97 ± 2.8500^d^
HD3	65.57 ± 3.2540	68.97 ± 2.3670	45.87 ± 0.8031^b^
HD4	66.87 ± 0.2216	70.42 ± 0.9413	39.58 ± 2.0280^a c^

^a^Indicates statistically significant differences between HD and EDB-1 based systems (P < 0.05).

^b^Indicates statistically significant differences between HD and EDB-1 based systems (P < 0.001).

^c^Indicates statistically significant differences between HD and EDB-2 based systems (P < 0.05).

^d^Indicates statistically significant differences between HD and EDB-2 based systems (P < 0.01).

Figure 15b shows the photopolymerization profiles with different photo-initiating systems, all of which reached maximum value of Rp (rate of photopolymerization) in 5 s and almost complete the photopolymerization in 10 s and the values of Rp_max_ are as following: HD1 ~ 36.99%, HD2 ~ 36.97%, HD3 ~ 45.89%, HD4 ~ 39.58% vs. EDB-1 ~ 31.74%, EDB-2 ~ 50.40%. Notably, HD3 and HD4 showed higher photopolymerization rate with Rp_max_ value up to 39.58–45.89% compared to EDB system (Rp_max_ 31.75%, P < 0.001, Table 1).

**Figure 17:**
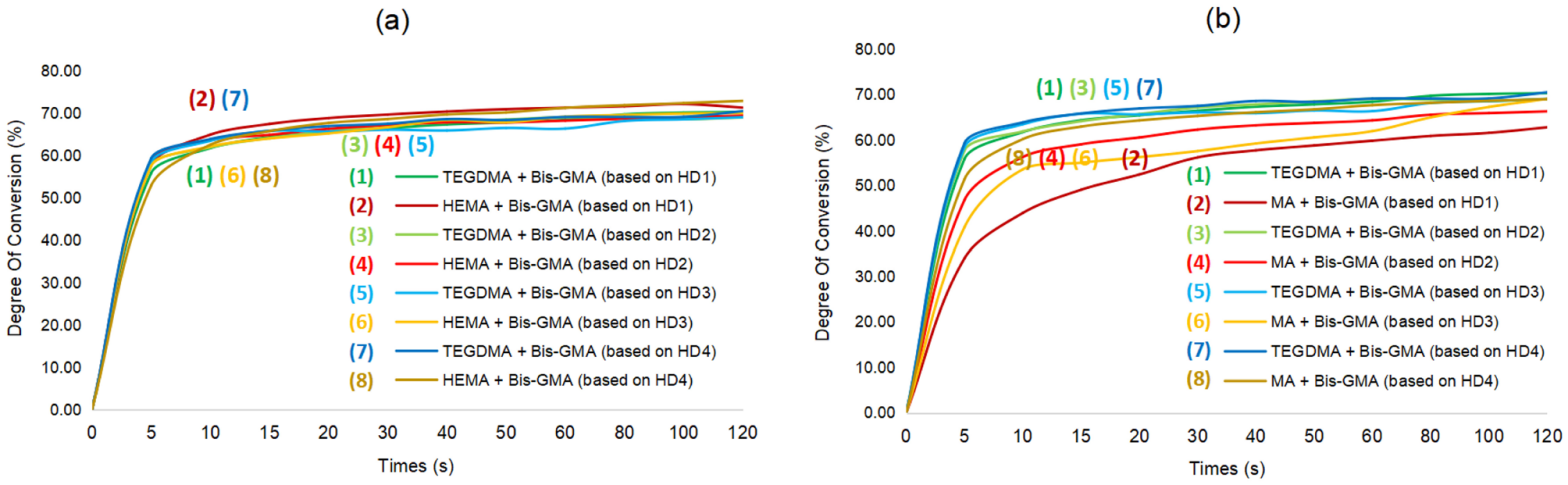
Photopolymerization profiles for different system based on lignin α-O-4 hydrogen donor. (**a**) Degree of C=C conversion vs. time of HEMA; (**b**) degree of C=C conversion vs. time of MA.

In order to investigate the solvent effect on the new photo-initiating systems, we examined photopolymerization performances using other monomers such as 2-hydroxyethyl methacrylate (HEMA) or methyl methacrylate (MA) instead of triethylene glycol dimethacrylate (TEGDMA). TEGDMA and HEMA with new HDs were comparable in terms of conversion, affording > 65% DC in 20 s and > 68% DC in 120 s (Fig. 17). However, formula using MA instead exhibited lower DC. Tables 2 and 3 suggested that no statistical difference was observed if the solvent was replaced with HEMA. However, when the solvent was replaced with MA, statistical differences were observed with HD1 and HD2 at 20 s and HD1 at 120 s, and their performance was inferior to that of HEMA. There was no statistical difference observed for the other groups.

**Table 2:** DC for different solvents based on different HD at 20 s.

	TEGDMA (mean ± SEM %)	HEMA (mean ± SEM %)	MA (mean ± SEM %)
HD1	65.38 ± 0.3341	68.77 ± 0.8637	52.34 ± 4.7900^a^
HD2	65.63 ± 0.21577	66.23 ± 0.3905	60.49 ± 1.0270^b^
HD3	65.57 ± 3.2540	65.15 ± 1.0450	56.15 ± 5.5450
HD4	66.87 ± 0.2216	67.61 ± 2.3790	64.23 ± 2.4460

^a^Indicates statistically significant differences between different solvents based on TEGDMA systems (P < 0.05).

^b^Indicates statistically significant differences between different solvents based on TEGDMA systems (P < 0.01).

**Table 3:** DC for different solvents based on different HDs at 120 s.

	TEGDMA (mean ± SEM %)	HEMA (mean ± SEM %)	MA (mean ± SEM %)
HD1	70.23 ± 0.7912	71.58 ± 1.1490	62.71 ± 1.9410^a^
HD2	68.91 ± 1.8110	69.34 ± 0.6637	66.24 ± 0.6536
HD3	68.97 ± 2.3670	69.97 ± 1.0300	68.98 ± 2.157
HD4	70.42 ± 0.9413	72.84 ± 2.0130	68.92 ± 2.2780

^a^Indicates statistically significant differences between different solvents based on TEGDMA systems (P < 0.05).

### Colorimetric measurements

For amine containing photo-initiating system, one of the issues is the residual color of the resin samples after photopolymerization, so the L*, b*, a* color indexes were measured to characterize the final color properties of the samples after photo-polymerization using novel co-initiators (Table 4). Statistical analysis of the b* values, which represent the yellow color, showed no significant difference between the experimental and control groups, indicating that the color bleaching effect of HDs was equivalent to that of the control group (Table 5: EDB-1-b* ~ 8.163, HD1-b* ~ 8.350, HD4-b* ~ 8.160, HD3-b* ~ 7.410, HD4-b* ~ 7.217, P > 0.05). It is noteworthy that the system based on EDB exhibits a visually higher degree of yellowness compared to the systems with HDs.

**Table 4:** Pictures of the mixed samples before and after fully cured in 1.4 mm thick molds.

	Chemical structure	Before	After
EDB-1			
HD1			
HD2			
HD3			
HD4

**Table 5:** Color indexes for the samples based on different photoinitiating systems (N = 3).

	Chemical structure	Color index(after irradiation)		
		L* (mean ± SEM)	a* (mean ± SEM)	b* (mean ± SEM)
EDB-1		86.25 ± 0.2307	− 2.023 ± 0.0636	8.163 ± 0.3294
HD1		84.44 ± 0.0895^a^	− 1.427 ± 0.0296^b^	8.350 ± 0.1752
HD2		85.37 ± 0.2237	− 1.257 ± 0.0817^c^	8.160 ± 0.7328
HD3		86.16 ± 0.3620	− 1.223 ± 0.0612^c^	7.410 ± 0.3573
HD4		85.67 ± 0.3620	− 0.9733 ± 0.1071^c^	7.217 ± 0.5136

^a^Indicates statistically significant differences between HDs and EDB based systems (P < 0.05).

^b^Indicates statistically significant differences between HDs and EDB based systems (P < 0.001).

^c^Indicates statistically significant differences between HDs and EDB based systems (P < 0.0001).

Above ageing test scenario was performed to measure color stability of disc specimens, and the formula of resin contains novel hydrogen donors HDs and EDB. Only unpigmented experimental samples were examined in this test. The ΔE value was determined to evaluate the color stability for each sample by conducting aging scenario (Table 6). The ΔE of HD1 (5.05), HD4 (4.10) in scenario 1 and HD2 (4.65), HD4 (3.83) in scenario 2 showed a higher ΔE compared to EDB system without Iod (P < 0.05), and the ΔE of other group showed no significant difference when compared to EDB system, showing comparable color stability than EDB in these scenarios.

**Table 6:** Results of ΔE measurements of the f the samples based on different photoinitiating systems.

	Scenario 1 (mean ± SEM)	Scenario 2 (mean ± SEM)	Scenario 3 a (mean ± SEM)	Scenario 3 b (mean ± SEM)
EDB	1.93 ± 0.0928	1.81 ± 0.2904	9.49 ± 0.1648	9.47 ± 0.5732
HD1	5.05 ± 0.4746^c^	2.93 ± 0.6113	10.09 ± 0.9833	10.40 ± 0.6667
HD2	2.58 ± 0.4218	4.65 ± 0.4460^b^	9.28 ± 0.3818	10.57 ± 0.2515
HD3	3.27 ± 0.5117	2.85 ± 0.3886	9.38 ± 0.6788	8.74 ± 0.2714
HD4	4.10 ± 0.0742^a^	3.83 ± 0.2340^a^	9.25 ± 0.4480	8.95 ± 0.3467

^a^Indicates statistically significant differences between HDs and EDB based systems (P < 0.05).

^b^Indicates statistically significant differences between HDs and EDB based systems (P < 0.01).

^c^Indicates statistically significant differences between HDs and EDB based systems (P < 0.001).

### Cytotoxicity evaluation

**Figure 18:**
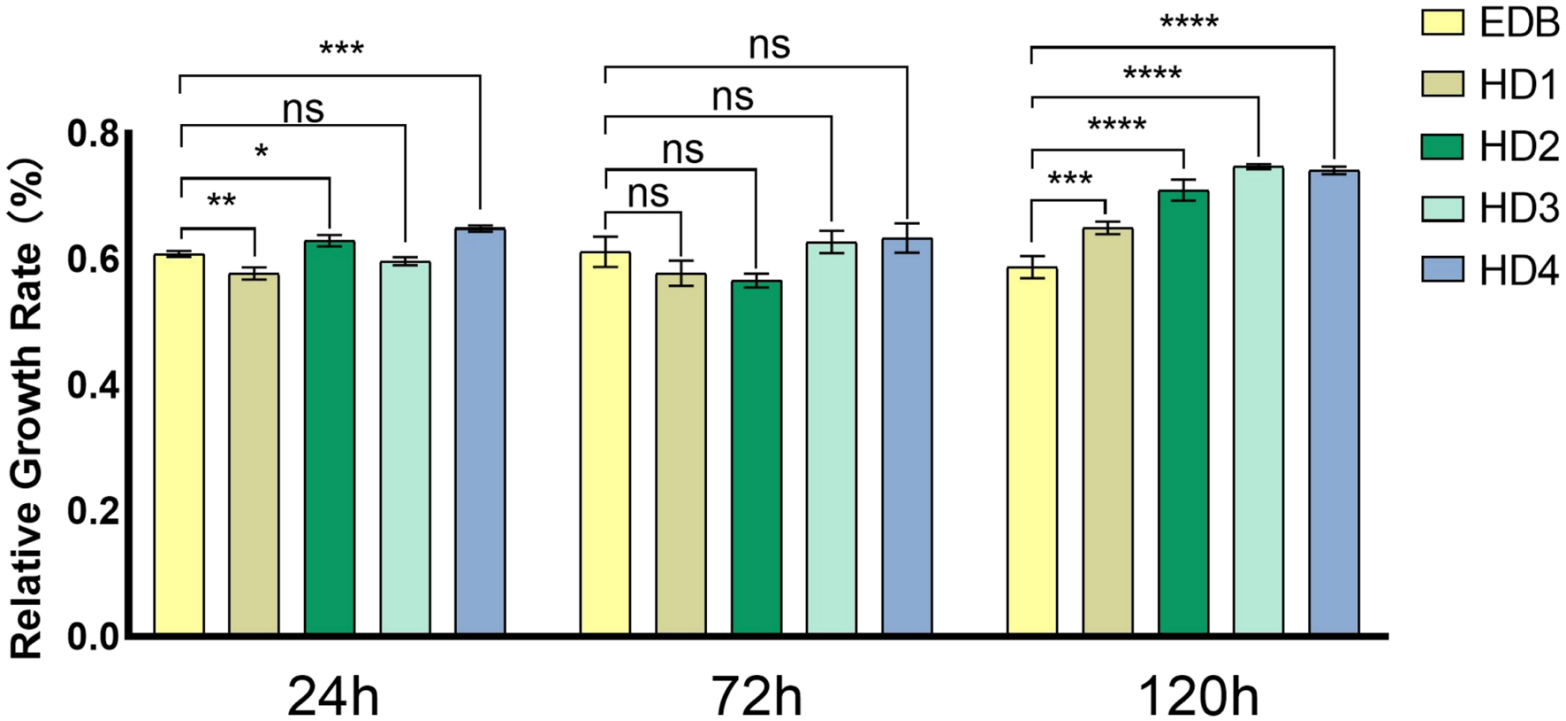
Cytotoxicity of specimens based on novel hydrogen donor, and compared with control group based on EDB (one-way ANOVA, “NS” represent no significant differences between each group; *P < 0.05; **P < 0.01; ***P < 0.001; ****P < 0.0001).

All experimental groups and control groups were carried out by CCK-8 cytotoxicity test. The cell viability was shown in Fig. 18. According to the ISO 10993-5 standard, both of the experimental and control groups were classified as exhibiting mild cytotoxicity (50% ≤ RGR < 100%. Table 7 shows that the data of the experiment groups showed significant statistical differences with control group at 120 h (P < 0.0001 for HD2, HD3 and HD4, P < 0.001 for HD1), suggesting that the cytotoxicity to be lower than that of the EDB. These results also exhibited all of the experimental groups to be similar to control group at 72 h.

**Table 7:** Relative growth rate (RGR) of HDs and EDB based systems.

	24 h (mean ± SEM)	72 h (mean ± SEM)	120 h (mean ± SEM)
EDB	0.6082 ± 0.004789	0.6117 ± 0.02430	0.5873 ± 0.01788
HD1	0.5773 ± 0.009676^b^	0.5777 ± 0.02032	0.6497 ± 0.01012^c^
HD2	0.6293 ± 0.008752^a^	0.5661 ± 0.01078	0.7098 ± 0.01673^d^
HD3	0.5965 ± 0.006233	0.6271 ± 0.01773	0.7469 ± 0.004066^d^
HD4	0.6489 ± 0.005090^c^	0.6337 ± 0.02308	0.7412 ± 0.006102^d^

^a^Indicates statistically significant differences between HDs and EDB based systems (P < 0.05).

^b^Indicates statistically significant differences between HDs and EDB based systems (P < 0.01).

^c^Indicates statistically significant differences between HDs and EDB based systems (P < 0.001).

^d^Indicates statistically significant differences between HDs and EDB based systems (P < 0.0001).

### Mechanical properties

The flexural strength of HD3 suggests 51.82 MPa, which is significantly lower than that of the control group EDB (Table 8). Similarly, the elastic modulus of HD2 suggests 1.263 GPa, which is significantly lower than that of the control group EDB. There are no statistically significant differences in the other mechanical strengths between the resins and the control group EDB.

**Table 8:** BDE of new hydrogen donor and compared to EDB.

	Flexural strength (mean ± SEM, MPa)	Elastic modulus (mean ± SEM, GPa)
EDB	78.77 ± 1.222	1.900 ± 0.0971
HD1	67.89 ± 3.123	1.933 ± 0.0267
HD2	67.89 ± 9.975	1.263 ± 0.0775^b^
HD3	51.82 ± 7.886^a^	1.627 ± 0.0921
HD4	59.34 ± 1.985	1.875 ± 0.1135

^a^Indicates statistically significant differences between HDs and EDB based systems (P < 0.05).

^b^Indicates statistically significant differences between HDs and EDB based systems (P < 0.01).

### Molecular modelling

The bond dissociation energy (BDE) of the compounds were calculated by molecular orbital calculations at the M06-2X1/6-311G (d, p) level (Table 9). The BDE of benzylic C-H bonds of new hydrogen donors are characterized by M06-2X1/6-311G (d, p) level calculation to be 82.80–84.14 kcal/mol, which are much lower than the N-methyl C–H bond of EDB (90.65 kcal/mol).

**Table 9:** BDE of new hydrogen donor and compared to EDB.

Compound	Chemical structure	BDE (kcal/mol)
EDB		90.65
HD1		84.14
HD2		83.95
HD3		82.80
HD4		83.87

## Discussion

The abundance of lignin in the natural environment, its ease of acquisition, and exceptional regenerative capabilities make it a promising candidate for sustainable material development and green chemistry. The wide-ranging prospects of lignin applications play a vital role in mitigating environmental burdens and promoting the sustainable utilization of resources.

We synthesized four lignin-derived hydrogen donors HD1, HD2, HD3 and HD4, which are firstly utilized as photo-initiation systems in photo-polymerization dental materials.

In this research, HDs, Iod, and CQ were used as ternary photo-initiation systems to afford ~ 70% DC in 120 s, which are comparable to CQ/EDB and CQ/EDB/Iod system. Herein, Iod salt is mandatory to enhance photopolymerization efficiency by providing an extra free radical and preventing CQ molecules from reverting to the stable state, which is supported by Piva’s research^27^. The new photo-initiating systems with HDs reached maximum value of Rp in 5 s and almost complete the photopolymerization in 10 s, showing their comparable and even higher polymerization rate than EDB system. The difference in polymerization efficiency can directly impact the curing process of dental resin. If the photoinitiator system induces a lower polymerization efficiency, it may lead to inadequate strength, poor sealing properties, or other performance issues during the curing process of dental resin. In this experimental section, HD3 exhibited the best performance.

Triethylene glycol dimethacrylate (TEGDMA) was replaced by other monomers such as 2-hydroxyethyl methacrylate (HEMA) or methyl methacrylate (MA) to examined the solvent effect. While HEMA gave comparable results in DC in 120 s, MA led to much lower DC presumably due to the existence of carboxylic acid to inhibit the photo-polymerization. These results exhibit that the new photo-initiating system was significantly influenced by the solvent type, and acidic solvent was unsuitable.

The color bleaching and color stability of the resin are also important standards for practical usage of the materials. The yellow color disappearance of camphorquinone during the light-curing process of dental resin is attributed to chemical reactions and bleaching processes, resulting in structural changes within the camphorquinone molecules, leading to a gradual loss of the yellow color and concurrently promoting the curing of dental resin. The variation in resin bleaching efficiency can be attributed to the different excitation efficiencies of hydrogen donors (HDs), thus implying that the composition of the photoinitiator system can significantly impact the bleaching performance of the resin. Statistically, the b values of the photo-initiation system based on HDs do not exhibit significant differences, indicating similar color bleaching performance between the photo-initiation based on HDs and EDB. However, the HDs system demonstrates visually superior bleaching performance compared to the EDB system. Among them, HD3 (b* ~ 7.410) and HD4 (b* ~ 7.217) demonstrate the most outstanding performance. The proper selection of hydrogen donors is crucial for enhancing the color stability and preventing color aging of dental resin materials. In the research and selection of dental materials, a comprehensive consideration of hydrogen donor stability, free radical reaction rate, degradation products, and other factors is essential to ensure that the materials exhibit excellent color stability during the light-curing process and long-term usage. HD1 and HD4 exhibit poorer color stability compared to EDB in Scenario 1, while HD2 and HD4 demonstrate inferior color stability to EDB in Scenario 2. In the remaining scenarios, the color stability of the HDs system is similar to that of EDB. It is noteworthy that HD3 gave similar results to the EDB system across all scenarios including water absorption and short-term light exposure. In this experimental section, HD1, HD2, and HD4 showed poor performance, while only HD3 demonstrated comparable results to EDB.

The biocompatibility of the resin is crucial for the further usage in dental materials. After 120 h of culture, the experimental groups showed a higher relative cell proliferation rate than that of the control group, indicating that the cytotoxicity of HDs/Iod/CQ photo-initiation system to be lower than the traditional photo-initiation system with EDB. The decreased toxicity of the novel photo-initiators further demonstrated their practical utility in the future.

In the performance testing of dental materials, mechanical properties are also very important indicators, such as elastic modulus and flexural strength. In the performance of flexural strength, EDB (78.77 MPa) > HD1 (67.89 MPa) ≈ HD2 (67.89 MPa) > HD4(59.34 MPa) > HD3(51.82 MPa). In the performance of Rp_max,_ EDB (31.75%) < HD1(36.99%) ≈ HD2(36.97%) < HD4(39.58%) < HD3(45.87%). Suggest that a higher polymerization rate of HDs and a lower flexural strength. While the elastic modulus has no significant correlation with photopolymerization. To improve mechanical properties, the inorganic fillers can be adjusted in later stages.

Molecular orbital calculations confirmed the benzylic C-H bonds of lignin-derived hydrogen donors (82.80–84.14 kcal/mol) to be much lower than N-methyl C-H bond of EDB (90.65 kcal/mol), suggesting a facile hydrogen abstraction from hydrogen donor to the triplet state of CQ, which are also in agreement with their good co-initiator behavior in photo-polymerization process. A pure hydrogen transfer step is expected as the new HDs are characterized by lower BDEs (C–H) than EDB, which ensure facile hydrogen abstraction reaction from the triplet state of camphorquinone^11^.

Oxygen molecules can react with the generated free radicals to form stable peroxides or peroxide radicals, which significantly reduces the number of free radicals to inhibit the polymerization process^1^. Gaviria-Martinez and colleagues found that the oxygen inhibition effect is primarily localized at the resin surface to form the so-called “oxygen-inhibited layer” (OIL) with decreased cure degree, however, polishing can effectively remove this OIL to enhance mechanical properties such as surface hardness, wear resistance and aesthetic outcomes of the resin^28^. In this experiment, the conversion rate rapidly decreased in the first 2 s, mainly due to the formation of an OIL on the surface of the samples during polymerization. Inert gas protection or addition of antioxidants could be considered to inhibit the oxygen inhibition effect but it will prevent the practical usage. In clinical applications, techniques such as polishing can effectively enhance the mechanical strength and aesthetic quality of light-cured resin fillings.

## Conclusions

In this work, four new co-initiators (HD1, HD2, HD3 and HD4) derived from α-O-4 lignin model were proposed. In comparison to *N*-methyl C–H bond dissociation energy of EDB, much lower benzyl C–H bond dissociation energies of the new hydrogen donors determined by molecular orbitals theory ensure their favorable hydrogen transfer with CQ and thus promote the photo-polymerization efficiently. The polymerization profiles using new co-initiators are at least similar to the sample using CQ/EDB system. Moreover, HD3 based amine-free photo-initiating system showed better Rp and cytotoxicity than the EDB based system. A content of 1% of HDs is required for the satisfactory photo-polymerization and end-color properties. Also, the decreased toxicity of the novel photo-initiators than the EDB further demonstrated their clinical safety. This research showed that the novel lignin α-O-4 derivative shows significant potential as a hydrogen donor in the initiation system for light-curing materials designed for dental applications, further extending its applicability in the field of dentistry.

### Supplementary Information

Supplementary Figures.

## Data Availability

The data that support the findings of this study are available on request from the corresponding author, Dr. Rui He, upon reasonable request.
