# Supplementary material for: Novel lignin α-O-4 derived hydrogen donors in CQ-based photoinitiating systems for dental resins
Source: Sci Rep. 2024 Jul 19;14:16719. doi: 10.1038/s41598-024-67377-z (PMC11271577; doi:10.1038/s41598-024-67377-z)
Supplement: Supplementary file 1 — Supplementary Figures. [file 41598_2024_67377_MOESM1_ESM.pdf]

Novel lignin α-O-4 derived hydrogen donors in CQ-based photoinitiating systems for dental resins

Lixia Xu[a,b#], Ying Zhang[a,e#], Shuqi Jin[c], Shuxin Luo[a,d], Kailun Chen[a,e], Sheng Fang[a,e], Liangjun Zhong[a,e], Jian Zhang[a,c,e*], Rui He[a,e*]

a. Center of Stomatology, The Affiliated Hospital of Hangzhou Normal University, Hangzhou, Zhejiang, China

b. The 3rd People’s Hospital of Deqing, Huzhou, Zhejiang, China

c. College of Material, Chemistry and Chemical Engineering, Key Laboratory of Organosilicon Chemistry and Material Technology, Ministry of Education, Hangzhou Normal University, Hangzhou, Zhejiang, China

d. Zhejiang Provincial Hospital of Chinese Medicine, Hangzhou, Zhejiang, China

e. School of Stomatology, Hangzhou Normal University, Hangzhou, Zhejiang, China

# These authors contributed equally to this work.

* These authors contributed equally to this work.

NMR Charts

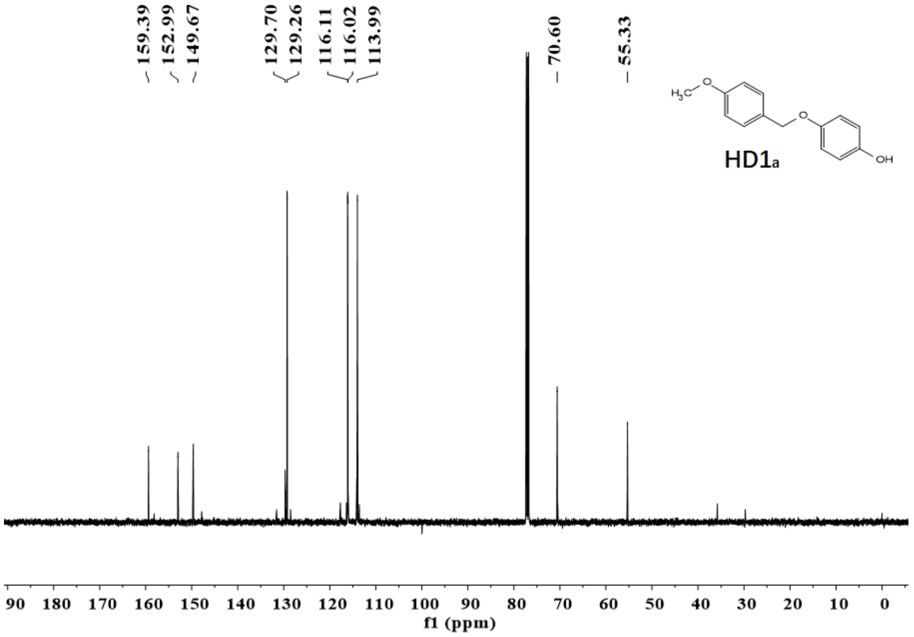

Supplementary Fig. 1 The 13C-NMR spectra of HD1a

^{13}C NMR (126 MHz, Chloroform-*d*) δ 159.39, 152.99, 149.67, 129.70, 129.26, 116.11, 116.02, 113.99, 70.60, 55.33.

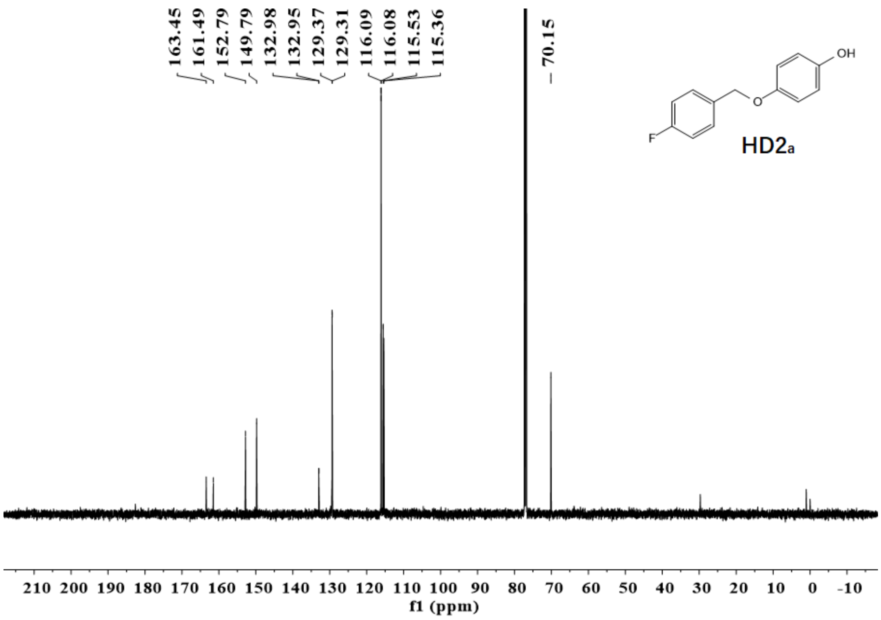

Supplementary Fig. 2 The 13C-NMR spectra of $HD2_a$

^{13}C NMR (126 MHz, Chloroform-*d*) δ 162.47, 152.79, 149.79, 132.96, 129.34, 116.08, 115.53, 115.36, 70.15.

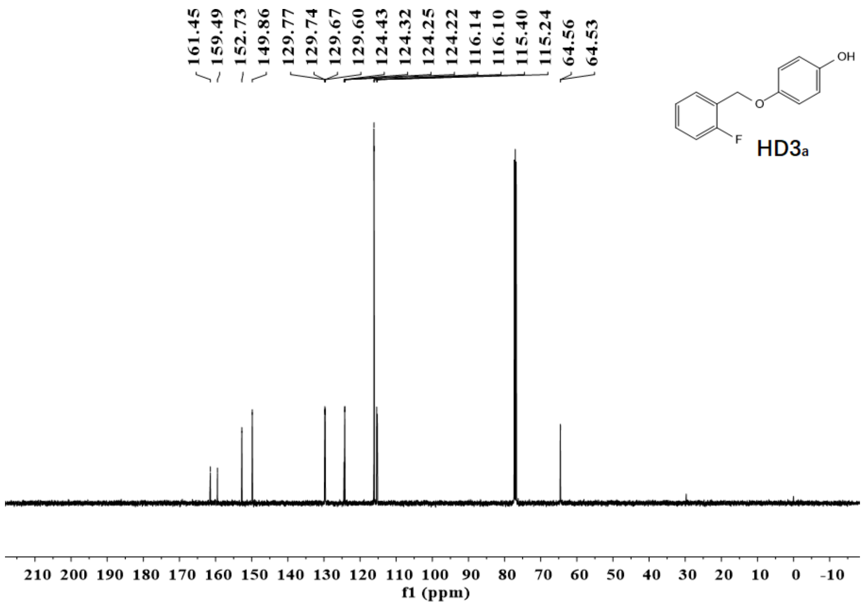

Supplementary Fig. 3 The 13C-NMR spectra of $HD3_a$

^{13}C NMR (126 MHz, Chloroform-*d*) δ 160.47, 152.73, 149.86, 129.75, 129.64, 124.38, 124.23, 116.12, 115.40, 115.24, 64.54.

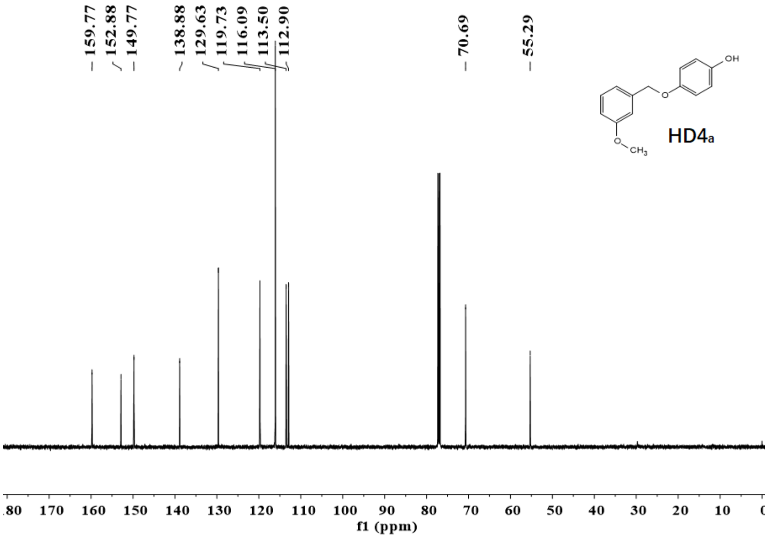

Supplementary Fig. 4 The 13C-NMR spectra of HD4a

^{13}C NMR (126 MHz, Chloroform-*d*) δ 159.77, 149.77, 138.88, 129.63, 119.73, 116.09, 116.07, 113.50, 112.90, 70.69, 55.29.

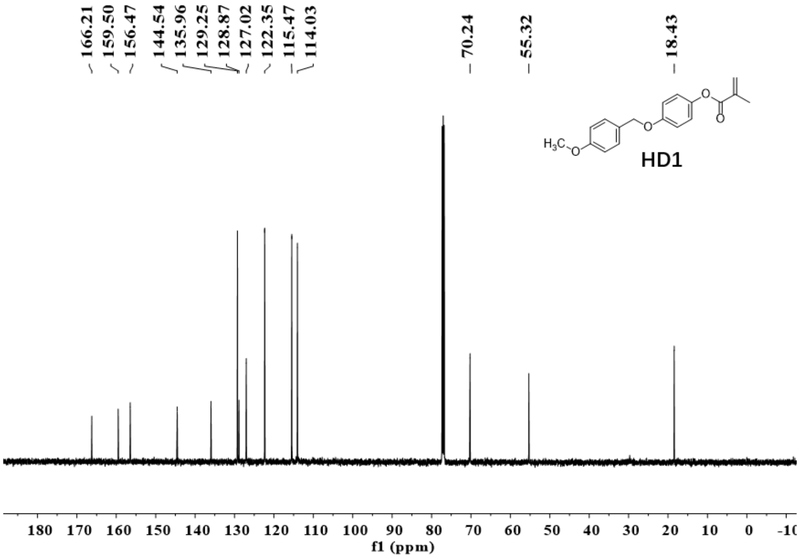

Supplementary Fig.5 The 13C-NMR spectra of HD1

^{13}C NMR (126 MHz, Chloroform-*d*) δ 166.21, 159.50, 156.47, 144.54, 135.96, 129.25, 128.87, 127.02, 122.35, 115.47, 114.03, 70.24, 55.32, 18.43.

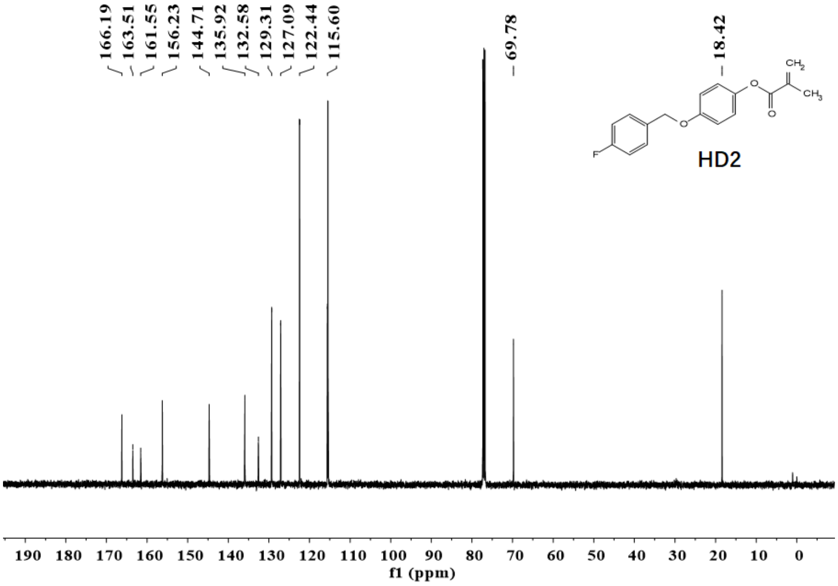

Supplementary Fig. 6 The 13C-NMR spectra of HD2

^{13}C NMR (126 MHz, Chloroform-*d*) δ 166.19, 162.53, 156.23, 144.71, 135.92, 132.58, 129.31, 127.09, 122.44, 115.60, 115.45, 69.78, 18.42.

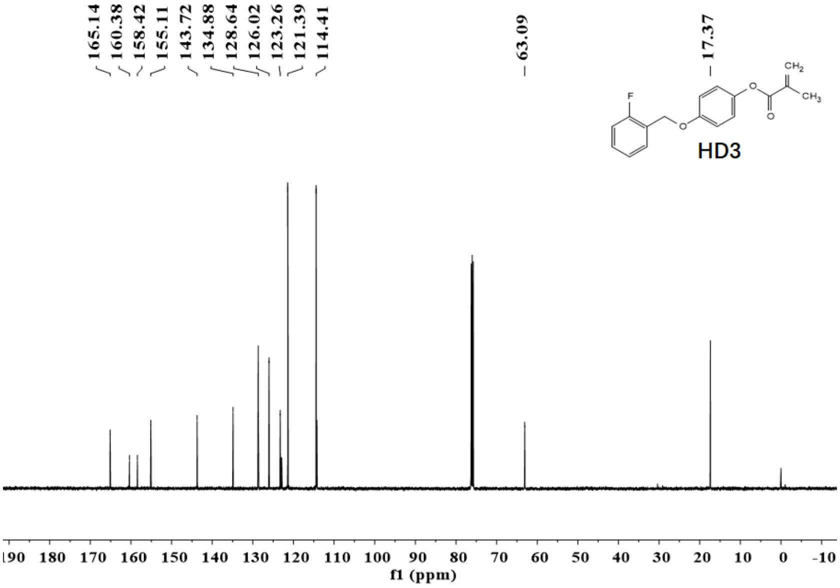

Supplementary Fig. 7 The 13C-NMR spectra of HD3

^{13}C NMR (126 MHz, Chloroform-*d*) δ 165.14, 159.40, 155.11, 143.72, 134.88, 128.74, 128.64, 126.02, 123.26, 122.97, 121.39, 114.41, 114.23, 63.09, 17.37.

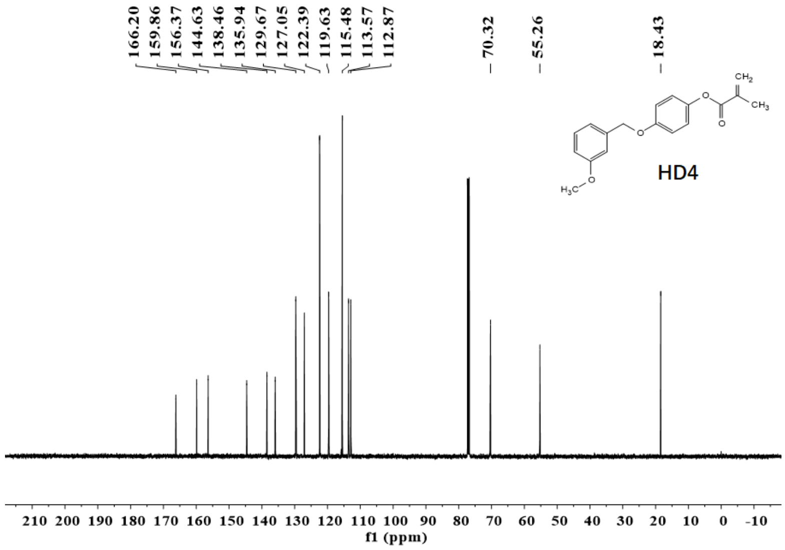

Supplementary Fig. 8 The 13C-NMR spectra of HD4

^{13}C NMR (126 MHz, Chloroform-*d*) δ 166.20, 159.86, 156.37, 144.63, 138.46, 135.94, 129.67, 127.05, 122.39, 119.63, 115.48, 113.57, 112.87, 70.32, 55.26, 18.43.
